# Factors affecting IGF-I level and correlation with growth response during growth hormone treatment in LG Growth Study

**DOI:** 10.1371/journal.pone.0252283

**Published:** 2021-07-19

**Authors:** Ji Hyun Kim, Su Jin Kim, Jieun Lee, Choong Ho Shin, Ji-Young Seo

**Affiliations:** 1 Department of Pediatrics, Dongguk University Ilsan Hospital, Goyang, Korea; 2 Department of Pediatrics, Inha University School of Medicine, Incheon, Korea; 3 Department of Pediatrics, Inje University Ilsan Paik Hospital, Goyang, Korea; 4 Department of Pediatrics, Seoul National University Children’s Hospital, Seoul National University College of Medicine, Seoul, Korea; 5 Department of Pediatrics, Eulji General Hospital, Eulji University College of Medicine, Seoul, Korea; University of Auckland, NEW ZEALAND

## Abstract

Growth hormone treatment strategies to achieve the goal include the titration of GH doses according to serum insulin-like growth factor I (IGF-I) concentrations. However, IGF-I levels do not always correlate well with the growth response. This study aims to identify the factors affecting the IGF-I concentration and identify the relationship between IGF-I and the treatment response. The data of prepubertal children treated with recombinant human GH for more than one year were obtained from the LG Growth Study (LGS) Database. This study includes patients with idiopathic growth hormone deficiency (IGHD), organic growth hormone deficiency (OGHD), or Turner syndrome (TS) or small for gestational age (SGA). Among 2,021 participants registered in LGS, 366 subjects were selected, 252 had IGHD, 16 had OGHD, 31 had TS, and 67 were SGA. In the IGHD and SGA groups, IGF-I levels had a positive correlation with weight SDS. There was no significant relationship between the pre-treatment IGF-I level and growth response. However, in the IGHD group, the growth response was significantly higher when the change in the IGF-I SDS value was 1 or more (*p* = 0.0013). Therefore, IGF-I concentrations should be used as an indicator to monitor the treatment compliance rather than for efficacy determination in Korean children of short stature with GH treatment.

## Introduction

Current growth hormone (GH) therapy assists many children undergoing treatment to achieve a final height close to the target height within the average age range. To realize this goal, many physicians personalize the growth effects of patients and adjust their GH dose. The therapeutic effect of GH is known to vary depending on the age of the patient and the rationale of treatment [1].

For this reason, various indicators have been proposed and used to determine the therapeutic effect of GH. The most frequently used indicators in clinical settings are auxological parameters, such as growth velocity and height SDS change. The most widely used biochemical markers are IGF-I and IGFBP-3. IGF-I is commonly used to determine GH treatment response, evaluate treatment compliance, and adjust a dose [2].

IGF-I also differs according to gender, body mass index, nutritional status, and puberty [3–5]. Furthermore, IGF-I levels vary according to the disease group, GHD is related to the severity of the disease [6], and SGA is related to insulin resistance [7]. Therefore, each study shows various resulting correlations between IGF-I and the growth response. Because the LG Growth Study (LGS) is a non-interventional registry study for Koreans, the IGF-I values can be compared with the same reference. In this study, we aim to identify factors affecting the IGF-I level for each disease group in prepubertal patients with short stature and determine whether there is a relation between IGF-I and the growth response.

## Material and methods

The data for this study was taken from the LGS. The LGS is a non-interventional registry study to evaluate the long-term safety and effectiveness of four recombinant human growth hormone (rhGH) products, three daily rhGH products (LG Chem Ltd., Korea; Eutropin®, Eutropin® AQ, and Eutropin® Pen), and one weekly rhGH product (Eutropin® Plus) used in Korea. The study was registered at ClinicalTrials.gov (identifier: NCT01604395) [8, 9]. The original LGS study included five cohort groups: growth hormone deficiency (GHD), idiopathic short stature (ISS), small for gestational age (SGA), Turner syndrome (TS), and chronic renal failure (CRF). When the GH stimulation test was indicated, appropriate tests were chosen among insulin-induced hypoglycemia, L-dopa, clonidine, L-arginine, or glucagon. After at least two GH stimulation tests, children with a peak GH level below 10 ng/mL [10] were classified as GHD in the LGS database. Children in this age group included in the LGS study performed a GH stimulation test without sex steroid priming. This study included three cohorts—GHD, SGA, and TS—and the GHD participants were reclassified into IGHD and OGHD. Among these, IGHD participants were classified into complete (<5 ng/ml) and partial (5–10 ng/ml) according to the peak GH concentration of the GH stimulation test. The OGHD group confirmed organic causes of GHD [8], and the GH peak was confirmed to be less than 10 through the GH stimulation test. We excluded the CRF group because various drugs affect IGF-I levels, and the ISS group, which could affect IGF-I levels due to heterogeneous causes, was also excluded.

All participants were prepubertal and had longitudinal treatment data, including auxology and serum IGF-I measurements from 9 to 15 months after the start of GH therapy. We included only subjects in the prepubertal stage at baseline and during the follow-up period. Puberty was determined based on the Tanner stage confirmed by a pediatric endocrinologist. For more clarity, at the first year of visit, boys with chronological age (CA) of 9 years or older and girls with a CA 7 years or older must check their bone age. All patients were treated for more than 1 year, and some continued for more than 3 years. Bone age (BA) was determined by the treating physician and was based on Greulich and Pyle’s standards. All anthropometric measurements were converted to z-scores using a Korean growth standard [11]. The IGF-1 SDS values were calculated using the standard Korean reference [12].

We analyzed the target group’s pre-treatment clinical characteristics, height, parent height, age, bone age, sex, height, weight, BMI, and each SDS, IGF-I, and IGF-I SDS before treatment. The height, body weight, and BMI were expressed as SDS values using the Korean growth standard based on chronologic age and gender. The mid-parental height (MPH) was calculated as (Mothers height + Fathers height)/2 ± 6.5cm and then expressed as SDS. For continuous data, the average, standard deviation, minimum, and maximum values were obtained. For categorical data, the frequency and the ratio were found.

To confirm the factors affecting the concentration of IGF-I according to the disease group, a correlation analysis between the demographic characteristics and the mean GH dose (mg/kg/week) was performed at the start of treatment. After one year of treatment, multiple regression analyses were performed to confirm the correlation between IGF-I SDS and the height SDS change. Change (Δ) in a variable induced by GH treatment was assessed as the difference between two values. Subsequently, treatment responses in the second and third years of treatment were established in the same manner. Additionally, after one year of treatment, IGF-I SDS changes were divided into subgroups of the high (**≥** 1) and the low (< 1), and treatment responses by disease were compared.

Written informed consent was obtained from patients and their parents/legal guardians prior to enrolment at each institute. The present study protocol was reviewed and approved by the Institutional Review Board of Dongguk University Ilsan Hospital (IRB no. DUIH 2020-04-008). This study was conducted in accordance with the Declaration of Helsinki. Statistical data analyses were performed using SAS® version 9.4 (SAS Institute, Inc., Cary, NC, USA). Multivariate analysis of variance was used to evaluate the factors associated with the IGF-I response.

## Results and discussion

**Fig 1:**
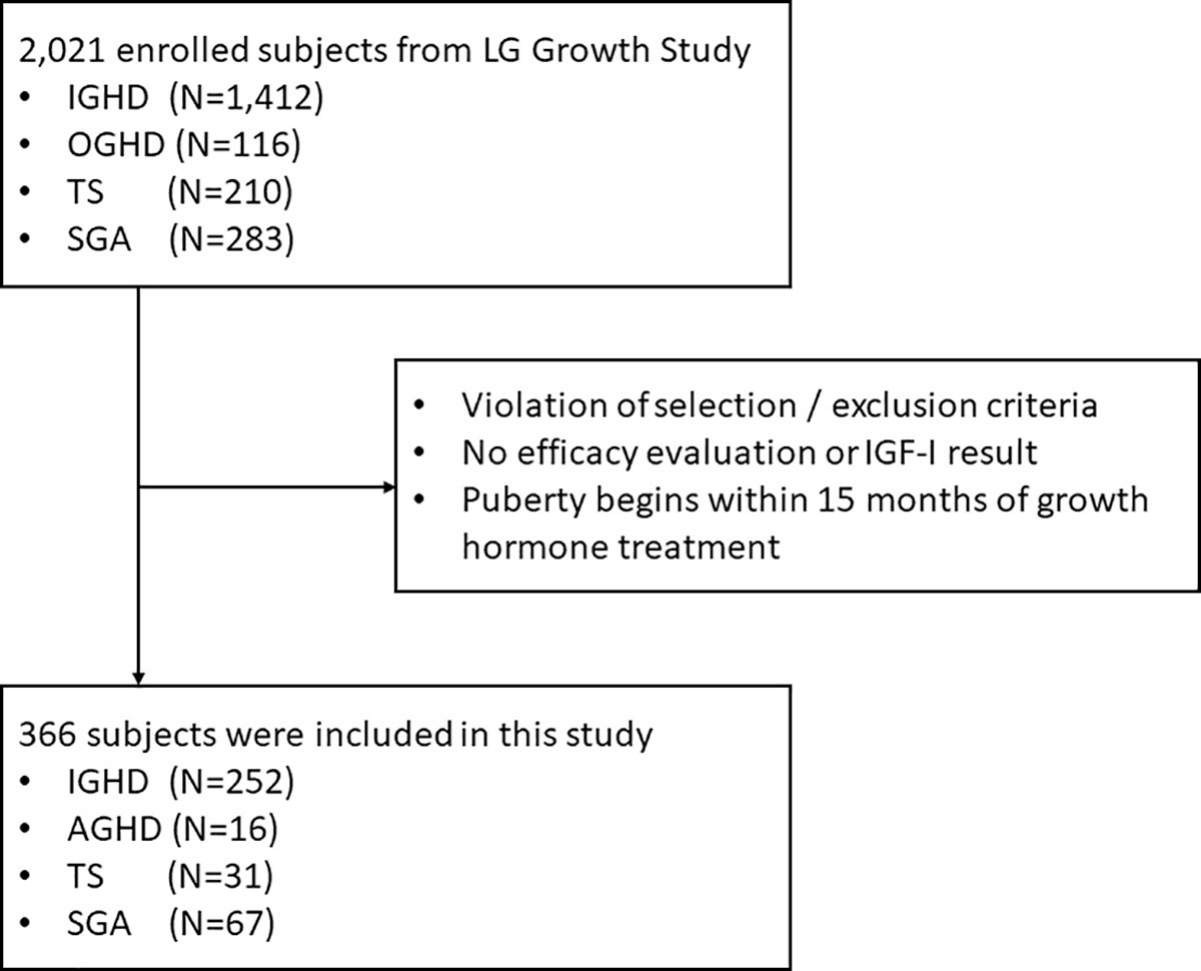
Flow chart of subject inclusion in this study.

In the LGS database, baseline auxology and serum IGF-I levels were available for 268 patients with GHD (IGHD: *n* = 252; OGHD: *n* = 16), 31 with TS, and 67 born SGA (Fig 1). The characteristics of the study subjects are summarized in Table 1. The MPH SDS did not differ between groups. In the GHD group, there was no statistically significant relationship between the GH stimulation test peak value and the pre-treatment IGF-I level. Lastly, the initial GH dose was 0.30 mg/kg/week, and there was no difference in each group.

**Table 1:** Demography at screening.

	IGHD (*n* = 252)		OGHD (*n* = 16)	TS (*n* = 31)	SGA (*n* = 67)
	Complete (*n* = 66)	Partial (*n* = 186)			
**Sex (male/female)**	49/17	121/65	11/5	0/31	38/29
**Age (years)**	6.14 ± 2.6	5.98 ± 2.24	7.38 ± 3.03	7.13 ± 2.95	5.61 ± 1.66
**Bone age (years)**	4.48 ± 2.27	4.49 ± 2.14	5.47 ± 2.76	6.52 ± 2.83	4.96 ± 1.81
**Height SDS**	-3.03 ± 0.97	-2.66 ± 0.55	-2.33 ± 1.75	-2.9 ± 0.75	-2.57 ± 0.54
**Weight SDS**	-2.16 ± 1.11	-1.93 ± 0.92	-1.07 ± 1.97	-1.62 ± 1.13	-2.32 ± 1.2
**BMI SDS**	-0.38 ± 1.15	-0.31 ± 1.11	0.35 ± 1.39	0.07 ± 1.09	-0.8 ± 1.27
**Mid-parental height (SDS)**					
** Male**	-0.71 ± 0.65	-0.75 ± 0.77	-0.33 (±0.90)	-	-0.81 (±0.44)
** Female**	-0.40 ± 0.93	-0.92 ± 0.71	0.43 (±0.63)	-0.24 (±0.86)	-1.02 (±0.62)
**IGF-I SDS**	-0.83 ± 1.20	-0.73 ± 0.88	-0.7 ± 2.41	-0.72 ± 0.85	-0.21 ± 1.32
**GH peak (ng/ml)**	3.40 ± 1.33	7.61 ± 1.49	2.8 ± 2.06		
**GH dose (mg/kg/week)**	0.35 ± 0.36	0.30 ± 0.23	0.29 ± 0.29	0.32 ± 0.04	0.26 ± 0.08

Values are presented as mean ± standard deviation unless otherwise indicated.

Abbreviations: IGHD; idiopathic growth hormone deficienc, OGHD; organic growth hormone deficiency, TS; Turner syndrome, SGA; small for gestational age, SDS; standard deviation score, BMI; body mass index, IGF-I; insulin-like growth factor-I, GH; growth hormone

Pre-treatment IGF-I values were associated with height SDS only in the complete IGHD. IGF-I concentration showed a positive correlation with weight SDS in the complete IGHD and SGA groups. IGF-I showed a negative correlation with the MPH in the SGA (Table 2). In other words, the taller and the greater the weight, the higher the IGF-I value in complete IGHD. The larger the weight and the shorter the parent’s height, the greater the pre-treatment IGF-I in the SGA.

**Table 2:** Correlations between the IGF-I concentration and screening measurements.

	IGF-I SDS at the screening				
	IGHD		OGHD	TS	SGA
	Complete	Partial			
**CA (years)**	-0.146^†^	-0.131	-0.151	0.202	0.024
**BA (years)**	-0.050	-0.068	0.006	0.369^*^	0.206
**MPH SDS**	-0.038	-0.167	0.036	-0.213	-0.320^*^
**Height SDS**	0.052^*^	0.023	-0.006	0.228	0.124
**Weight SDS**	0.455^*^	0.141	-0.168	0.275	0.434^*^
**BMI SDS**	0.173	0.115	0.000	0.123	0.393^*^
**GH dose (mg/kg/week)**	0.264	0.013	-0.291	-0.281	0.144

^†^Spearman correlation coefficient

*p-value < 0.05

Abbreviations: CA; chronologic age, BA; bone age, MPH; mid-parental height, SDS; standard deviation score, BMI; body mass index, GH; growth hormone

After one year of treatment, the treatment response was evaluated by the change in height SDS. The OGHD group was excluded because the number of subjects was too small to calculate statistical analyses through multivariate regression. Pre-treatment factors influencing changes in height SDS during the first year of treatment was identified only in the IGHD group.

A significant change in height SDS was associated with smaller height SDS at treatment in complete IGHD (*r* = -0.5597, *p* = 0.0286), a higher MPH in partial IGHD (*r* = 0.0878, *p* = 0.0348; Table 3). The GH dose did not show a significant relationship with the growth response. According to multiple regression analysis, IGF-I SDS was not associated with the growth response after one year of treatment.

**Table 3:** Multiple regression analyses between the height response and screening measurements one year after treatment.

	△ Height SDS (1 year–Screening)			
(Screening)	Complete IGHD	Partial IGHD	TS	SGA
**IGF-1 SDS**	0.078^†^	0.005	0.046	-0.027
**Height SDS**	-0.560*	-0.079	-0.011	-0.081
**Weight SDS**	0.272	0.080	-0.186	0.247
**BMI SDS**	-0.073	-0.033	0.308	-0.128
**MPH SDS**	0.119	0.088*	-0.033	-0.036
**CA (years)**	-0.100	-0.085	0.085	-0.178
**BA (years)**	-0.006	0.010	-0.119	0.063
**Sex**	-0.048	0.062	0	0.001
**Total GH Dose (mg/kg/week)**^**§**^	-0.006	<0.001	0.005	0.001
***R-Square***	0.782	0.289	0.417	0.292

OGHD was excluded because the number of subjects was too small to perform statistical analysis through multiple regression.

^§^Total GH dose (mg/kg/week): sum of the dosage from screening to 1 year

^†^Regression coefficient

*p-value < 0.05

Abbreviations: IGHD; idiopathic growth hormone deficiency, TS; Turner syndrome, SGA; small for gestational age, IGF-I; insulin-like growth factor-I, SDS; standard deviation score, BMI; body mass index, MPH; mid-parental height, CA; chronologic age, BA; bone age, GH; growth hormone

After one year of GH treatment, the clinical characteristics of high and low IGF-I responses were compared across all groups. An increase of more than one in IGF-I SDS was calculated as 51.5% for complete IGHD, 59.7% for partial IGHD, 57.5% for IGHD, 62.5% for OGHD, 67.7% for TS, and 74.6% for SGA. In the partial IGHD groups, the high IGF-I subgroup (SDS ≥ 1) had a higher pretreatment height SDS (*p* = 0.0237) and a larger weight SDS (*p* = 0.0273) and body mass index (BMI) SDS (*p* = 0.0291) than the low IGF-I (SDS < 1) subgroup. There was no difference in △IGF-I SDS and peak GH values in both complete and partial IGHD groups (3.40 vs. 3.41 in complete; 7.68 vs. 7.51 in partial IGHD). In the TS group, there were no statistically significant differences between the low and high subgroups. Moreover, in the SGA group, the high IGF-I subgroup was greater BMI SDS (*p* = 0.0086).

**Fig 2:**
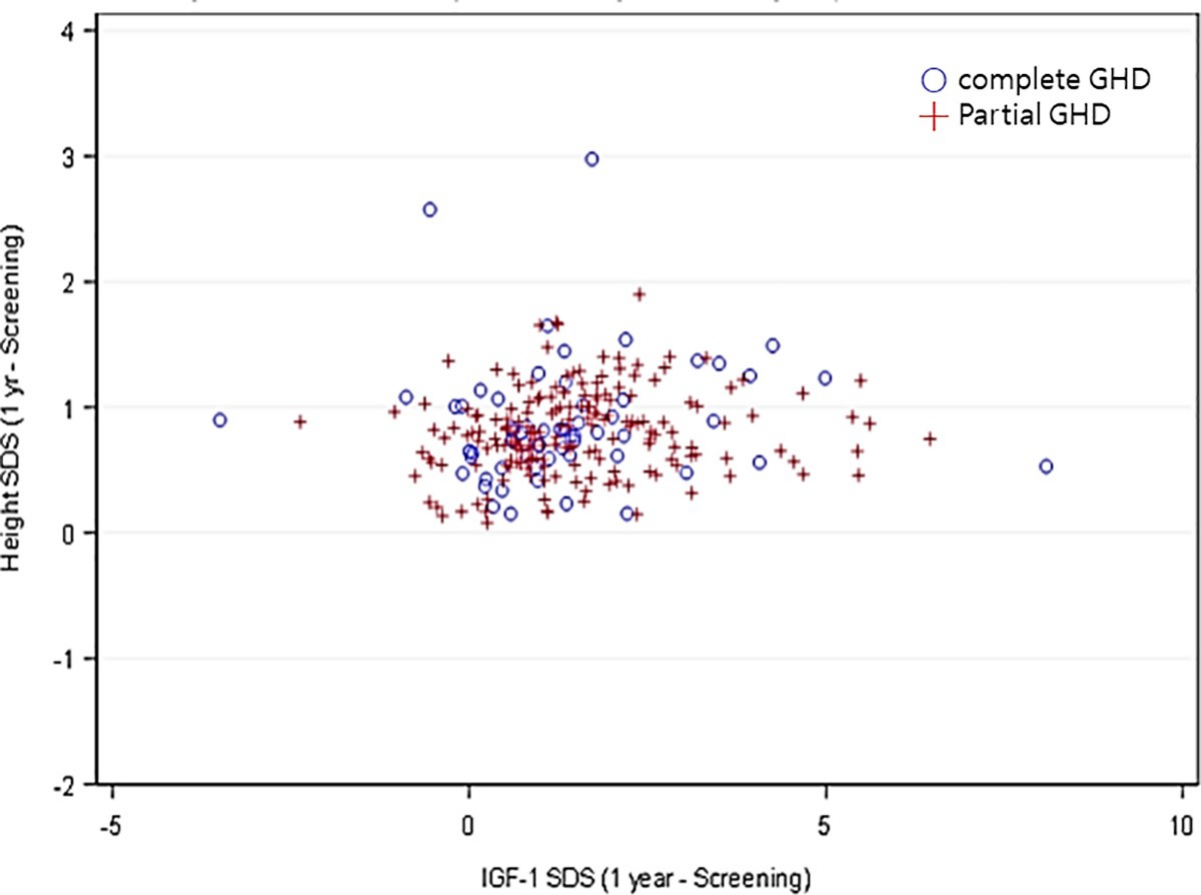
The first-year growth response and change of IGF-I SDS in the IGHD.

Furthermore, only the IGHD group showed weak positive correlations (Spearman correlations) between the first-year growth response and the △IGF-I SDS (*r* = 0.1918, *p* = 0.0022; Fig 2). In complete IGHD, height SDS increased by 1.0 in the high IGF-I subgroup in the first year after treatment (*p* = 0.0383; 0.78 in the low group) and 0.49 in the second year (*p* = 0.0169; 0.34 in the low group). In partial IGHD, height SDS increased by 0.86 in the high IGF-I subgroup in the first year after treatment (*p* = 0.0083; 0.73 in the low group) and 0.39 in the second year (*p* = 0.0406; 0.31 in the low group; Table 4).

**Table 4:** Height response group by △ IGF-1 SDS.

	Complete IGHD		Partial IGHD		TS		SGA	
	△IGF SDS ≥ 1 *n* = 34	△IGF SDS < 1 *n* = 32	△IGF SDS ≥ 1 *n* = 111	△IGF SDS < 1 *n* = 75	△IGF SDS ≥ 1 *n* = 21	△IGF SDS < 1 *n* = 10	△IGF SDS ≥ 1 *n* = 50	△IGF SDS < 1 *n* = 17
**△Height SDS**								
1 year–Screening	1.00 ± 0.54	0.78 ± 0.44	0.86 ± 0.35	0.73 ± 0.29	0.54 ± 0.08	0.56 ± 0.12	0.9 ± 0.06	0.84 ± 0.09
	*p* = 0.038*		*p* = 0.008*		*p* = 0.992		*p* = 0.383	
2 years–1 year	0.49 ± 0.29	0.34 ± 0.36	0.39 ± 0.23	0.31 ± 0.20	0.24 ± 0.06	0.14 ± 0.09	0.38 ± 0.05	0.37 ± 0.1
	*p* = 0.017*		*p* = 0.041*		*p* = 0.191		*p* = 0.599	
3 years–2 years	0.22 ± 0.25	0.21 ± 0.28	0.29 ± 0.25	0.22 ± 0.21	0.22 ± 0.05	0.07 ± 0.09	0.09 ± 0.22	0.21 ± 0.33
	*p* = 0.958		*p* = 0.501		*p* = 0.083		*p* = 0.712	

Values are presented as LS mean ± SE and *p*-value obtained from the ANCOVA model

*p-value < 0.05

Abbreviations: IGHD; idiopathic growth hormone deficiency, IGF-I; insulin-like growth factor-I, SDS; standard deviation score

After three years of treatment, there was no significant relationship between the degree of IGF-1 change and height SDS. Moreover, the TS and SGA groups did not show this correlation.

Our study identified the factors that affect IGF-I levels before treatment differs by disease group. Substantially pre-treatment IGF-I values and growth responses were not correlated; only the IGHD group showed a weak positive correlation between the growth response and change in the IGF-I value after one year of treatment. As far as the authors know, this study presents a well-documented result using standardized IGF-I values in a single country.

IGF-I is a major regulator of the growth and metabolism in the fetus and throughout childhood [13]. It also plays an essential role in the growth and development of skeletal muscle through systemic or local regulation of growth plates [14, 15]. Furthermore, IGF-I has been implicated as a key endocrine regulator of fetal and postnatal growth modified by glucose availability and insulin secretion in utero, nutrition in infancy, and GH during childhood [16]. During puberty, IGF-I levels rise to 2–3 times that of adults, which is well associated with the Tanner stage [3, 17]. The liver mainly generates circulating IGF-I with levels according to the protein intake degree, suggesting that higher protein intake stimulates IGF-I secretion [5].

Moreover, it has been found in studies that the serum IGF-I level is lower in children with malnutrition or GH deficiency [18, 19]. Additionally, height SDS, weight SDS, BMI SDS, total body fat, and fat-free tissue were positively correlated with the IGF-I. After adjusting for gender and current weight, IGF-I levels have been inversely related to birth size [20]. In the Avon Longitudinal Study of Parents and Children, low birth weight and robust catch-up in the first two years were associated with greater BMI and higher IGF-I levels at 5 years of age [7, 21]. Taller and obese children had 30% and 80% greater IGF-I responses to GH stimulation, respectively, compared to children of average stature [22, 23]. In our study, the association between weight SDS and the IGF-I level was found in the IGHD and SGA groups.

The association between IGF-I levels and GH status is well established. Many studies have demonstrated that GH action is associated with linear skeletal growth through IGF-I. In this regard, studies have shown a positive correlation between the height and growth effects of IGF-I levels in prepubertal GHD and non-GHD children [24–28]. Given that GH is the primary factor in determining circulating IGF-I levels, there is a debate about whether the higher IGF-I levels yield more significant growth effects. Also, even when GHD is diagnosed, the IGF-I value does not accurately reflect the GH status. Since only severe GHD shows low IGF-I values, it is recommended to perform a GH stimulation test for confirmation [10]. For instance, Bright et al. demonstrated that rhGH therapy results in a positive correlation between IGF-I levels and a two-year adjusted change in height SDS scores [24]. In other studies, IGF-I measurements are less helpful in adjusting the required GH dose. Still, they may help monitor compliance and safety when treating prepubertal GHD children with growth hormone [29–32]. Therefore serum IGF-I monitoring is not always related to growth response.

Thus, the assessment of growth response is the most critical parameter of treatment effectiveness. The growth response consists of height velocity and a height gain [33, 34]. During the initial two years of treatment, catch-up growth at a twofold to fourfold rate above the pre-treatment GV and an increase of 1–2 height SDS should be expected in the majority of patients [33, 35]. In the KIGS (Pfizer International Growth Database), the GH concentration and IGF-I levels were weakly related and should be used primarily for determining compliance [36]. The authors also stated that IGF-I monitoring would help clinicians judge when to increase GH doses. Our study, the IGHD group, showed a weak positive correlation between growth response and IGF value after correcting weight SDS. This result was more clearly observed in the complete IGHD group. Recently, as the GH test’s accuracy has improved, there are reports that the partial GHD be considered as ISS [37]. Our study tried to limit the subjects to prepubertal children, but sex steroid priming was not used in the GH stimulation test, so care should be taken in interpreting and applying partial IGHD results.

In order to confirm the IGF-I and growth response, IGF-I measurements should be standardized, and there should be little missing data. Variation in IGF-I levels with diurnal fluctuations and monthly intra-individual differences contribute to the difficulty in demonstrating clinically relevant as well as statistically significant differences [38]. However, like all registry studies, our study had some limitations. First, many patients were excluded due to incomplete data registration or missing measurement values during the follow-up period. Second, it was not possible to confirm and reflect on the time for measuring IGF-I. Third, the GH doses administered to TS and SGA were less than the usual dose. Since this study is data for 3 years of initial treatment, it is necessary to confirm the GH dose and treatment effect for these groups through long-term follow-up. Nevertheless, our study is meaningful because it demonstrated the relationship between the IGF level and growth response in a relatively large number of patients, excluding missing data, in a single country with the same criteria of IGF-I.

## Conclusions

We identified the factors influencing IGF-I concentration and found differences in disease groups. In IGHD, IGF-I changes were weakly associated with treatment effects, but they were no longer significant from the third year of treatment. Therefore, care should be taken in judging the therapeutic effect using the IGF-I concentration during GH treatment, and the influence of body weight should be considered. IGF-I concentrations should be used as an indicator to monitor treatment compliance rather than for efficacy determination.
